# A computational approach for modeling the biological olfactory system during an odor discrimination task using spiking neuron

**DOI:** 10.1186/1471-2202-12-S1-P360

**Published:** 2011-07-18

**Authors:** Roberto A Vázquez

**Affiliations:** 1 Intelligent systems group, Universidad La Salle, Mexico City, D. F., 06140, Mexico

## 

The biological olfactory system is capable of solving problems related to the olfactory information processing such as odor discrimination. This system is composed of three main parts: the layer receptors of the nose, the bulb and the piriform cortex [1]. The sense of smell is a chemical neural process where odorant molecules stimulate the olfactory system. These molecules are inhaled through the nose, where they contact the olfactory receptor neurons. The olfactory neurons transduce receptor activation into electrical signals in neurons. The signals travel along the olfactory nerve which terminates in the olfactory bulb. Finally, the olfactory bulb, which is composed of different cell layers, sends the information to the piriform cortex where discrimination between odors is performed. Odors are represented as patterns of neuronal activity. This representation may be encoded by space, time or a combination of both [2]. In this research, we described an approach for modeling the olfactory system in order to perform an odor discrimination task by means of the neural activity produced by a network of spiking neurons (SNs).

Our model is composed of three layers. The first layer contains a set of neurons acting as receptive fields, which normalized the input stimulus and sent the information to the next layer. The second layer is modeled with a linear-type neuron, which received the information from each receptor neuron and enhances sensitivity to odor and discrimination through adjusting the synaptic connections. After that, obtained signal is directly injected to the third layer, which is composed of a network of SNs. During learning phase, synaptic connections are adjusted by means of an evolutionary learning approach [3]. Finally, discrimination between odors is performed by means of the neural activity recorded in the SNs. The described model, allow us to discriminate odors using the neural activity encoded by time or space. In the first case, SNs fire at similar firing rates with odors from the same class; on the other hand, odors from different classes provoke SNs fire at a firing rate different enough to discriminate among the odors. In the second case, a set of activated SNs determines an olfactory region which corresponds to the odor.

To test the accuracy of the model during odor discrimination task a dataset was used [4]. This dataset contains 124 samples of two different classes of alcohol: butanol and ethanol. The dataset was split into training and testing dataset. During learning, the model performs with an accuracy of 96% and 92% in terms of the neuronal activity encoded by time and space, respectively. During testing, the model performs with 92% and 87%, respectively.

The model learnt to discriminate odors by means of the neural activity encoded by time and space. Successful results suggest that the model could serve as a biological model to explain the process of odor discrimination in the olfactory system.

**Figure 1:**
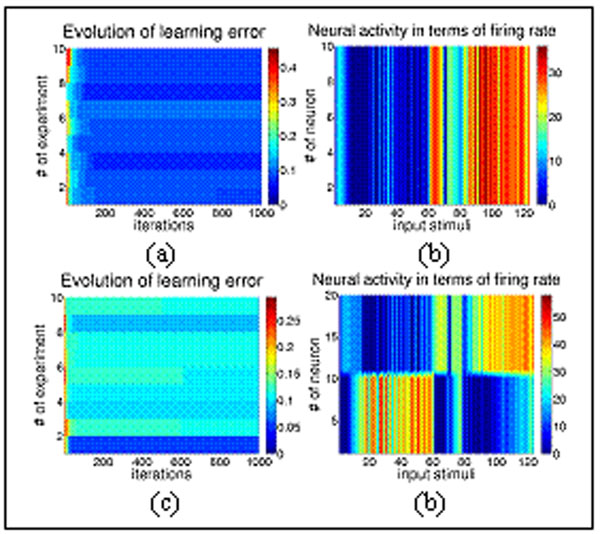
Learning error and neural activity during an odor discrimination task. (a) – (b) Neural activity encoded by time. (c) – (d) Neural activity encoded by space.
